# The reduction in the biomass of cyanobacterial N_2_ fixer and the biological pump in the Northwestern Pacific Ocean

**DOI:** 10.1038/srep41810

**Published:** 2017-02-03

**Authors:** Dongseon Kim, Jin-Hyun Jeong, Tae-Wook Kim, Jae Hoon Noh, Hyung Jeek Kim, Dong Han Choi, Eung Kim, Dongchull Jeon

**Affiliations:** 1 Marine Chemistry and Geochemistry Research Center, Korea Institute of Ocean Science & Technology, Ansan, South Korea; 2 Department of Marine Science, Incheon National University, Incheon, South Korea; 3 Marine Ecosystem and Biological Research Center, Korea Institute of Ocean Science & Technology, Ansan, South Korea; 4 Deep-sea and Seabed Mineral Resources Research Center, Korea Institute of Ocean Science & Technology, Ansan, South Korea; 5 Marine Safety Research Center, Korea Institute of Ocean Science & Technology, Ansan, South Korea; 6 Physical Oceanography Research Center, Korea Institute of Ocean Science & Technology, Ansan, South Korea

## Abstract

The comparison of sediment trap data with physical and biogeochemical variables in the surface water column of the Tropical Northwestern Pacific Ocean (TNWPO) indicated that the magnitude of the springtime biological pump has reduced with time due to a corresponding decrease in the biomass of cyanobacterial N_2_ fixer. The decrease in the biomass of N_2_ fixer likely resulted from a reduction in phosphate concentrations in response to surface water warming and consequent shoaling of the mixed layer depth during the study period (2009−2014). The same reduction in biological pump was also observed during summer. However, the cause of the summer reduction remains uncertain and is worth assessing in future studies. Our findings have major implications for predicting future trends of the biological pump in the TNWPO, where significant warming has occurred.

Phytoplankton living in the lighted upper ocean account for approximately a half of global primary production^1^,^2^. This conversion of inorganic carbon to organic matter entails the transport of CO_2_ from the atmosphere to the ocean interior because it reduces partial pressure of CO_2_ in the surface ocean (thereby facilitating air-to-sea transfer of CO_2_), and forms solid particles that may sink to the deep ocean. These phytoplankton-mediated processes are referred to as the biological pump, and have been regarded as a long term regulating mechanism for climate change occurring due to variations in atmospheric CO_2_.^2^ Intensive field surveys conducted during the past several decades have greatly advanced our knowledge on the air-sea exchange of CO_2_ in the global surface ocean, and enabled us to make quantitative estimates of the oceanic uptake of CO_2_.^3^ However, sparse observations of vertical particle flux in time and space have limited our understanding of the factors controlling this carbon sink^4^. As a result, currently it is difficult to accurately predict the response of the biological pump to global climate change^5^.

Recent studies suggest that phytoplankton productivity, and hence the magnitude of the biological pump, will be reduced in the future ocean because of depressed entrainment of nutrients under enhanced stratification^6^,^7^. However, N_2_ fixation occurring in relatively warm (>25 °C) oligotrophic waters may relieve this nutrient limitation because N_2_ fixation transforms inert N_2_ gas to bioavailable nitrogen which is a primary limiting nutrient for phytoplankton productivity in most oceanic environments^8^. Then N_2_ fixation will be one of key processes to determine the biological pump in the future ocean. To accurately assess this role of N_2_ fixation and have an integrated estimation over highly diverse oceanic settings (with varying environmental and meteorological conditions), the oceanic response of N_2_ fixation to climate change must be investigated in as many ocean regions as possible.

One of important areas requiring such investigations is the TNWPO, because environmental conditions (e.g., sea surface temperature and nutrient concentration) are rapidly changing^9^,^10^ and N_2_ fixation contributes considerably to primary productivity in this region^11^,^12^. Therefore we chose it as a study area, and investigated the relation between sinking particle flux (SPF) and N_2_ fixer biomass, and compared the results with ocean physical and biogeochemical parameters. Time series SPF data were obtained from particles collected using a sediment trap installed at a depth of ~1000 m (13.5°N, 135°E; bottom depth of ~5100 m, Fig. 1) from July 2009 to May 2014. Biogeochemical data (phytoplankton abundance, chlorophyll-*a* and nutrient concentrations) were collected from the upper water column of the mooring station, once a year (either May or June) for the 2009−2014 period. However, because our water column data were limited to only one season, we also used data collected by the Japan Meteorological Agency (JMA, data available at http://www.data.jma.go.jp/gmd/kaiyou/db/vessel_obs/data-report/html/ship/ship_e.php) to evaluate seasonal variations in the study area (this dataset was used for only this purpose).

## Results

The five-year mean fluxes (±1 standard deviation) of total mass flux (TMF), organic carbon (OC), calcium carbonate (CaCO_3_) and biogenic silica (BSi) were 9.88 ± 5.10, 0.78 ± 0.48, 6.74 ± 3.51 and 0.28 ± 0.27 mg m^−2^ d^−1^, respectively (Fig. 2). Consistent with previous results obtained in subtropical Pacific waters^13^,^14^ CaCO_3_ fluxes were higher than OC and BSi fluxes. Monthly mean TMF values varied seasonally (Fig. 3d). The TMF was usually greatest during the summer (July to September, ~12.99 mg m^−2^ d^−1^), followed by autumn (October to December, ~10.09 mg m^−2^ d^−1^), spring (April to June, ~8.24 mg m^−2^ d^−1^) and winter (January to March, ~8.09 mg m^−2^ d^−1^). However, the summer peak almost disappeared in 2011, and has since become even less distinct (Fig. 2). Similar annual decreases (from ~12.92 mg m^−2^ d^−1^ in 2010 to ~4.82 mg m^−2^ d^−1^ in 2014) were also observed during the spring months, whereas no significant trend was seen for autumn and winter (Figs 2 and 4). In addition, all three biological SPF components (OC, CaCO_3_ and BSi) decreased in the spring and the summer.

The springtime field survey revealed that biomass of phytoplankton and nutrient inventory simultaneously decreased with time. The abundance of *Crocosphaera watsonii* decreased from 2009 to 2014 (Fig. 5a) and the chlorophyll-*a* inventory and the abundance of *Prochlorococcus* and photosynthetic picoeukaryotes also decreased for the same period (Fig. 5b and c). Phosphate concentration was at least five times higher than its analytical detection limit (~0.01 μmol kg^−1^) of our flow injection autoanalyzer whereas most of nitrate samples collected within euphotic layer were either very close to or below the analytical detect limit (~0.1 μmol kg^−1^), which confirmed nitrate depletion in our study area. During 2009−2013, we observed a reduction in the phosphate inventory of the upper 100 m (Fig. 5d). The δ^15^N values (see method for notation) of our sediment trap samples were generally lower (4.25‰ as an average, Fig. 3e) than the values (5~6)^15^ in deep water nitrate.

## Discussion

**Figure 1:**
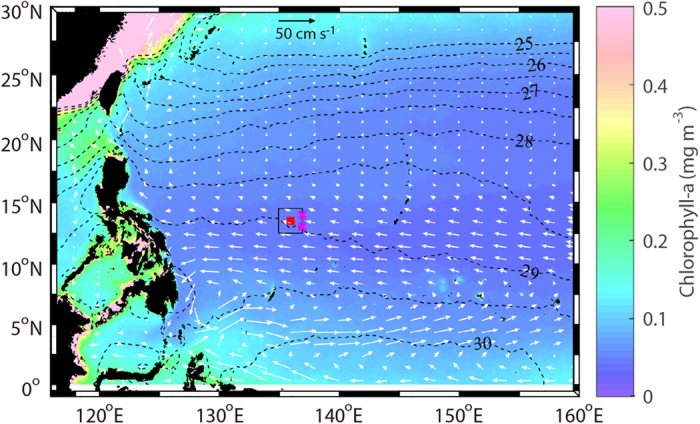
Mean surface water properties for the six year period (2009−2014). Color shading and contours represent chlorophyll-*a* concentration and sea surface temperature, respectively (MODIS aqua). White arrows indicate current flow directions (NCEP). The red circle (at 13.5°N, 136°E) in the center of the blank rectangle (+1° from the red circle) indicates the sediment trap mooring site. Magenta ‘×’ symbols stand for the JMA stations whose data were used in the study. This map was generated using Matlab^®^ R2015b (MathWorks Inc., http://www.mathworks.com/) and M_Map toolbox 1.4 h (https://www.eoas.ubc.ca/~rich/map.html).

**Figure 2:**
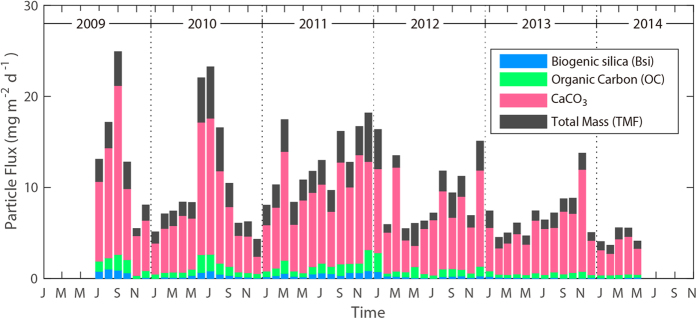
Time series of monthly averages of the total mass flux (TMF, black), CaCO_3_ flux (red), organic carbon flux (OC, green), and biogenic silica flux (BSi, blue) measured from July 2009 to May 2014.

**Figure 3:**
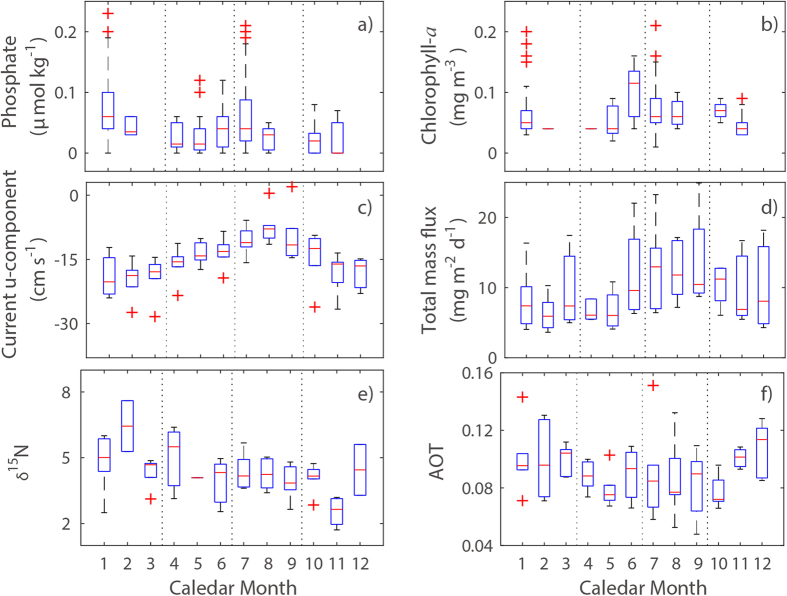
(**a**−**f**) Mean monthly variations within the blank-rectangle region (see Fig. 1) of (**a**) phosphate (JMA, 1970−2015); (**b**) chlorophyll-*a* (JMA, 1970−2015); (**c**) U-component of current (NCEP, 2009−2014); (**d**) total mass flux (this study, 2009−2014); (**e**) δ^15^N (this study, 2009−2014); and (**f**) aerosol optical thickness (MODIS, 2009−2014). All available JMA data during the given period were used to better construct the seasonal variations in phosphate and chlorophyll-*a*. Nonetheless, there were still no data for March, September, and December.

The seasonal pattern of SPF (especially summer peak) was broadly consistent with the seasonality in chlorophyll-*a* and phosphate concentrations measured in the surface layer (0−30 m) of the nearby JMA stations (Fig. 3a,b and d, see Fig. 1 for locations), suggesting a coupling between biogeochemical processes in the surface ocean and the SPF to the deep ocean. Therefore, the observed reduction in the biogenic components of the SPF suggested that there were significant changes in phytoplankton production in the overlying surface ocean, where productivity was largely controlled by new nitrogen supplied by N_2_ fixation^16^,^17^. The δ^15^N values of our sediment trap samples confirmed that N_2_ fixation (producing organic matters with δ^15^N = ~1)^18^ contributed considerably to ocean productivity in the study area. Therefore, it was likely that the observed temporal reduction in the SPF was associated with N_2_ fixation, which was supported by the reduction in the springtime abundance of *Crocosphaera watsonii* from 2009 to 2014 (Fig. 5a). From independent study conducted in 2008 for the same study area, we observed that the biomass of *Crocosphaera* spp. was three times greater than that of *Trichodesmium* spp. (see the method section), indicating *Crocosphaera* spp. is the dominant N_2_ fixation cyanobacteria in the study area. Although we had no measurement for diatom-diazotroph associations, their abundance and contribution to N_2_ fixation appeared to be reduced, inferring from the weakened BSi flux^19^.

**Figure 4:**
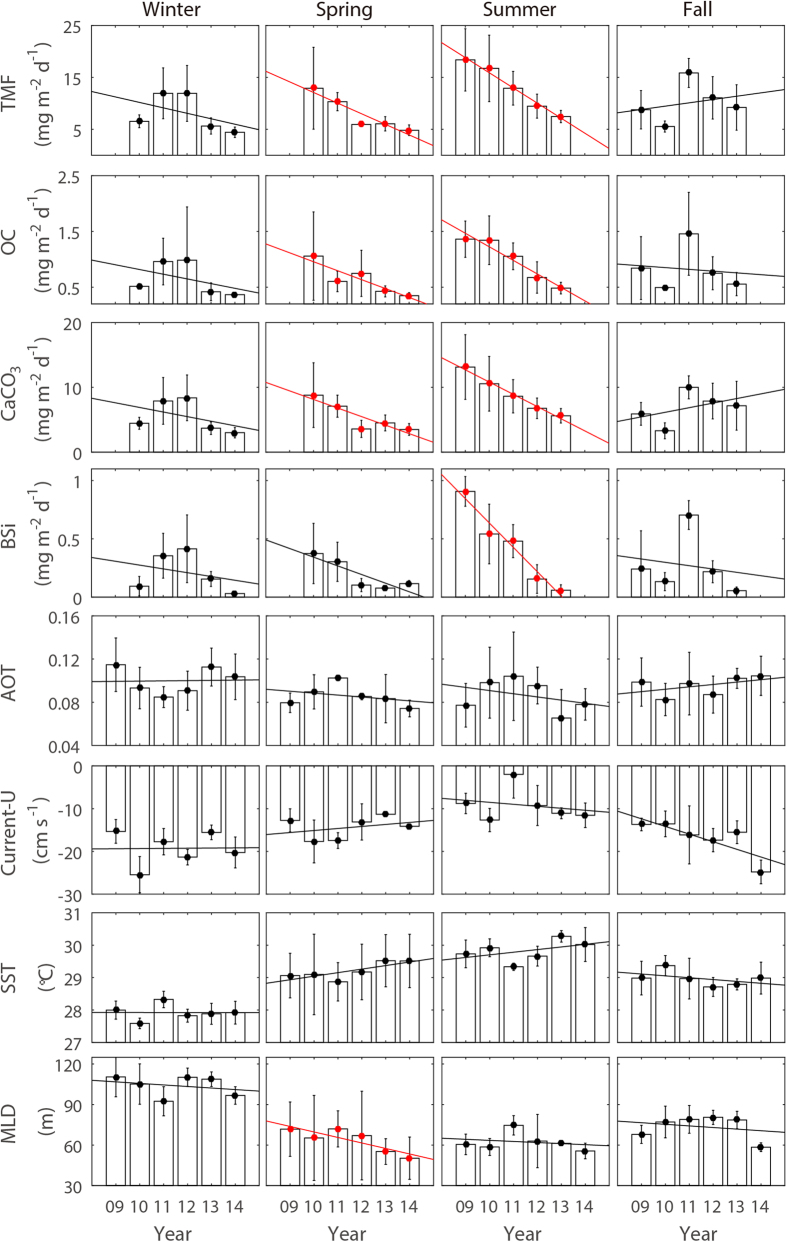
Interannual variations of seasonal averages (closed circles) of the TMF (total mass flux), OC (organic carbon), CaCO_3_ and BSi (biogenic silica) fluxes (mg m^−2^ d^−1^) and aerosol optical thickness (AOT, dimensionless), U-component of current (Current-U, cm s^−1^), sea surface temperature (SST, °C), and mixed layer depth (MLD, m) for the 2009−2014 period. Error bars indicate one standard deviation from the mean. Solid lines are regression lines. Red indicates that the temporal decrease is significant (*p* < 0.05).

The growth of N_2_-fixing microorganism depends on several factors, among which temperature and nutrient availability (iron, nitrogen and phosphorus) are known to be the most critical^20^. In the study area, the sea surface temperature (SST) was favorable for N_2_ fixation during the entire year (27~31 °C, Fig. 1). So, temperature *per se* could not constrain the growth of N_2_ fixers. Although we did not directly evaluate iron concentrations in the atmosphere and water column, it was possible to assess the temporal change in atmospheric iron input by using satellite-based estimates (aerosol optical thickness, AOT) of atmospheric aerosol/dust concentrations, provided by the Moderate Resolution Imaging Spectroradiometer (MODIS) aqua (data available at http://oceancolor.gsfc.nasa.gov/). No noticeable trends in the AOT were seen during the study period (Fig. 4). In addition, monthly variations were not coupled to variations in chlorophyll-*a*, phosphate, or the TMF (Fig. 3). More importantly, prevailing wind in the study area blows from the central Pacific Ocean. Therefore, atmospheric deposition of iron was not a main driver for the change in N_2_ fixer abundance, but the availability of macro-nutrients such as nitrogen and phosphorus (rather than iron) was likely to be the main driver for the observed reduction in the abundance of N_2_ fixers.

**Figure 5:**
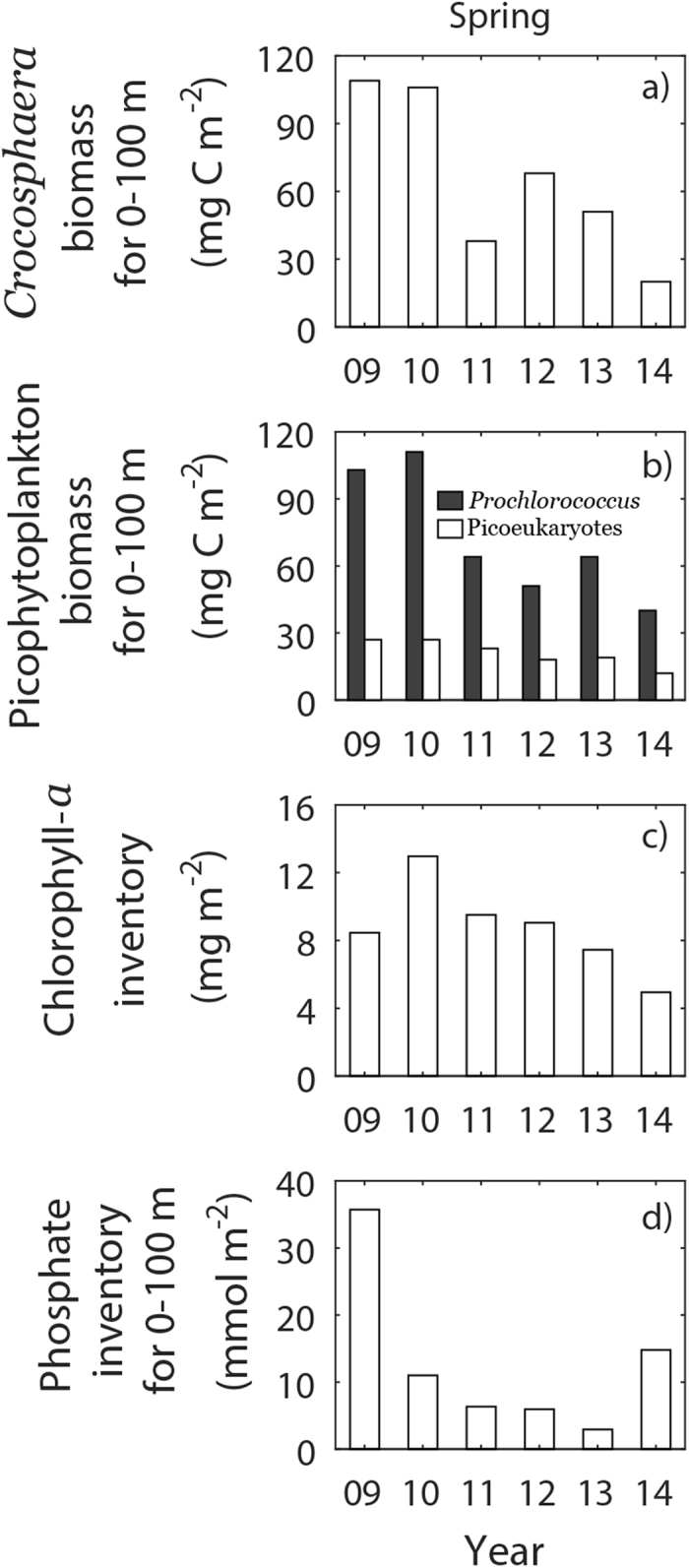
Interannual variations in the depth-integrated biomass (mg C m^−2^) of (**a**) *Crocosphaera* and (**b**) picophytoplankton (*Prochlorococcus* and Picoeukaryote), and the depth-integrated inventory of (**c**) chlorophyll-*a* (mg m^−2^), and (**d**) phosphate (mmol m^−2^). The depth-integration was done for the upper 100 m. All data were collected during spring.

Biological data collected during spring rejected the increase in nitrogen as a main driver for the changes in the SPF and N_2_ fixers. The chlorophyll-*a* inventory and the abundance of *Prochlorococcus* and photosynthetic picoeukaryotes decreased during the years when N_2_ fixer abundance declined (Fig. 5). Because N_2_ fixers require energy to convert N_2_ gas to a bioavailable form^20^, N_2_ fixers cannot compete with non-N_2_-fixing phytoplankton unless ambient levels of bioavailable nitrogen are low^11^. If bioavailable nitrogen concentrations had increased in the study area, *Prochlorococcus* and photosynthetic picoeukaryotes would have outcompeted N_2_ fixers. Therefore, it is unlikely that any nitrogen enhancement (via atmospheric deposition or vertical entrainment) occurred during the study period. Recent studies reported N_2_ fixation in nitrate-replete environment^21^,^22^. However, two studies conducted in the Western North Pacific Ocean reported no detectable N_2_ fixation in nitrate-replete waters^11^,^23^. In contrast to bioavailable nitrogen, phosphate (PO_4_^3−^) stimulates N_2_ fixation in seawater. Thus, an increase in phosphate concentrations was most unlikely.

Then again, a decrease in bioavailable nitrogen alone (without any concurrent changes in phosphate) cannot explain the diminishing N_2_ fixation that we observed, because depletion of bioavailable nitrogen favors the growth of N_2_-fixing cyanobacteria. Hence, a reduction in phosphate levels seems the most plausible cause for the reductions in the SPF and N_2_ fixation at the study site. This hypothesis is supported by previous studies that showed that the relative abundance of phosphate over nitrate was a primary constraint for N_2_ fixation in the subtropical and tropical Pacific Ocean^24^,^25^. Indeed, we observed a reduction in the phosphate inventory during the 2009−2013 spring surveys (Fig. 5d). Unfortunately, in 2014 showing a sharp increase in phosphate inventory, data on the corresponding SPFs were not available. We further investigated which of the phosphate sources weakened during the study period. Given that the study site was located far (>1200 km) from land, river influence was minimal, and atmospheric phosphorus deposition rates were probably low^12^,^26^,^27^. Given that these external phosphorus sources were negligible at the study site, we examined two ocean internal sources: horizontal current and vertical mixing.

The North Equatorial Current (NEC), a prevailing westward-flowing current in the TNWPO, appears to transport the most oligotrophic waters of the TNWPO to the west and dilute surface waters at the study site. The zonal chlorophyll-*a* distribution obtained from the MODIS aqua data clearly showed a gradual eastward decrease in concentration along the NEC flow pathway (Figure S1). Given the coupling of chlorophyll-*a* and nutrient concentrations (Fig. 3a and b), it seems likely that nutrients were distributed in a longitudinal pattern similar to that of chlorophyll-*a*. Thus, the intensity of the NEC would affect the availability of nutrients at the study site. Indeed, this association was apparent when seasonal variations were examined. Phosphate concentrations peaked in the summer months, when westward flows of the NEC were slowest (Fig. 3a and c). But in spite of its importance in determining the spatiotemporal pattern of nutrients, it appeared that the NEC did not play a decisive role in the reduction of the SPF during the course of the study. The interannual variations of the NEC were inconsistent with those of the SPF (Fig. 4). For example, mean springtime westward velocities seemed to decrease over the course of the study; this decrease should have reduced dilution and created phosphate concentrations favorable to N_2_ fixation.

The mixed layer depth (MLD) data provided by the National Centers for Environmental Prediction (NCEP) reanalysis project^28^ (data provided from http://www.esrl.noaa.gov/psd/) showed that the springtime mean MLD shoaled significantly from ~70 m to ~50 m between 2009 and 2014 (*p* < 0.05, Fig. 4). The NECP MLD was defined as the depth where the temperature deviation from SST is less than 0.8 °C. This shoaling may have alleviated phosphate supply from deeper waters, thereby causing reductions in both cyanobacterial N_2_ fixation and the SPF. However, a coherent temporal pattern between the MLD and the SPF was not observed during the summers (from 2009 to 2013). The NCEP MLD data were compared with the Argo-based MLD estimates provided by the Japan Agency for Marine-Earth Science and Technology (JAMSTEC)^29^. The two MLD data sets were significantly correlated (*r* = 0.76, Figure S2), suggesting that the NCEP MLD estimates for the study site were reliable. Note that the JAMSTEC data were only available when Argo floats acquired vertical profiles; hence, these data could not be used for the time-series analysis.

The SST affects nutrient availability in surface waters by influencing water column stability and consequently, nutrient upwelling. Watanabe *et al*.^9^ demonstrated that surface nutrient and chlorophyll-*a* concentrations were reduced in the TNWPO in response to a concurrent increase in the SST (but without a change in the MLD), based on an analysis of JMA data (collected between 3−15°N, along 137°E) during the 1971−2000 period. In our study area, mean SSTs in the spring and the summer seemed to increase from 2009 to 2014; but this increase lacked statistical significance (*p* = 0.065 and 0.35, respectively).

No remarkable difference was observed during a rapid transition from El Niño to La Niña between 2009 and 2010. The Pacific Decadal Oscillation changes in multi-decadal intervals. Hence, despite the critical roles of these two climate oscillations in biogeochemical cycles in the tropical Pacific Ocean^9^,^30^,^31^, we think they did not have a prominent impact on the reduction in the biological pump that we observed.

In summary, the reduction in the biological pump (SPF) in the TNWPO, was only noticeable during relatively warm seasons (spring and summer). Our annual springtime field surveys showed that the dwindling abundance of the dominant N_2_ fixer and the resulting reduction in the supply of new nitrogen to other phytoplankton were responsible for the weakened biological pump. The changes in the SST and the MLD might trigger the reduced supply of phosphate to surface waters in the spring and the summer. However, the changes were insignificant except for the MLD during spring. Thus, the cause for the reduction in summer SFP remains uncertain and is worth assessing in future studies. During the cooler months of autumn and winter, the water column was relatively well mixed (i.e. the SST was lower and the MLD was deeper), so the biological pump was not significantly affected. Continuing enhancement in water column stratification and nutrient limitation will cause the reduction in total phytoplankton production, diatom fractions and zooplankton biomass, eventually weakening export efficiency^32^. In addition, a change in the remineralization length scale induced by surface warming may also reduce the export of organic carbon through sinking 33.

Although our results were confined to five years at one site, they have broad implications in terms of the response of the biological pump to climate change (particularly, surface ocean warming^6^,^9^,^34^). The response of N_2_ fixation to enhanced stratification in our study area differed from that observed at the North Pacific subtropical gyre. Karl, *et al*.^35^ showed that N_2_ fixation had been enhanced during a period of prolonged stratification at Station ALOHA of the Hawaiian Ocean Time-series. Thus, it seems likely that responses may vary considerably across the global ocean. Hence it is vital that we conduct more studies, in as many oceanic settings as possible, to evaluate the response of N_2_ fixation and the biological pump to environmental changes. It is imperative to better understand spatial and temporal variations in these processes, if we wish to accurately predict their impact in the future, particularly in light of global climate change.

## Method

### Sediment trap data

Each collection cup of the sediment trap (McLane PARAFLUX Mark 78H-21) was filled with a formalin-poisoned solution made of sodium borate and filtered seawater. The depth recorder show that the sediment traps were positioned at the water depth of ~950 m for the first and third deployment years and ~1070 m for the remaining years. During the study period, current speeds were mostly less than 15 cm s^−1^ for the entire period, tilting by current would have minimal effects on trapping efficiency^36^. Recovered samples were immediately stored in a refrigerator (at 4 °C). The sampling interval was approximately 18 days. Each trap sample was split into five equal aliquots for multiple chemical analyses, using a McLane Wet Sample Divider-10. Four of the five aliquots were rinsed with deionized distilled water (*Milli-Q* water) to remove any residual formalin solution and sea salts. The rinsed samples were then freeze-dried and weighed to measure the total mass flux. The dried samples were homogenized in an agate mortar to determine total carbon, inorganic carbon, and biogenic silica content. Total carbon content was analyzed using an elemental analyzer (*Carlo-Erba 1110* CNS), and analytical error was maintained at < 3% using standard reference material (Sulfanilamide, *CE Instruments*). Inorganic carbon content was measured (at an accuracy of >98%) using a carbon analyzer (*UIC coulometrics)*. Calcium carbonate (CaCO_3_) content was calculated by multiplying the inorganic carbon content by a conversion factor of 8.33. Organic carbon content was estimated as the difference between total carbon and inorganic carbon. The resulting uncertainty of organic carbon determination approached ~25% due to relatively large amount of total carbon and CaCO_3_. Biogenic silica content was measured using a wet alkaline extraction method (with a precision of ~5%). If sample quantities were sufficient (>20 μg N) after determining the aforementioned parameters, the ratio of stable nitrogen isotopes (δ^15^N (%) = [(^15^N/^14^N)_sample_/(^15^N/^14^N)_atmN_ −1] × 1000, where the atmN indicates atmospheric N_2_) was analyzed in the US Davis Stable Isotope Facility.

### Field survey (2009–2014)

Seawater samples were collected within the euphotic zone, using a rosette sampler with Niskin bottles. Chlorophyll-*a* concentrations were determined using a Turner fluorometer (10 AU, Turner Design). Abundances of picocyanobacteria, picoeukaryotes and *Crocosphaera* were enumerated using a Beckman-Coulter Altra Flow Cytometer^37^, and *Crocosphaera* cell abundance was confirmed with epifluorescence microscopy. Cell numbers were converted to carbon biomass using conversion factors of 59 fg C cell^−1^ for *Prochlorococcus*^38^, 433 (cell volume)^0.863^ fg C cell^−1^ for pico-eukaryotes^39^ and 20 pg C cell^−1^ for *Crocosphaera*^40^. Phosphate concentrations were measured using a flow injection autoanalyzer (model QuikChem AE, Lachat, Loveland, CO, USA) and calibrated using brine standard solutions (CSK Standard Solutions, Wako Pure Chemical Industries, Osaka, Japan).

### Dominant N_2_ fixing species

We estimated cell abundance and biomass of two major types of diazotrophic cyanobacteria, *Trichodesmium* spp., and *Crocosphaera* spp. in 2008 at the study area. Depth-integrated cell abundance of *Trichodesmium* spp. and *Crocosphaera* spp. were approximately 1.7 × 10^7^ trichomes m^−2^ and 68.7 × 10^10^ cells m^−2^, respectively. Conversion factors used for these two cyanobacteria biomasses were 30,000 pg C trichome^−1^ and 20 pg C cell^−1^, respectively^40^. The results indicated that carbon biomass of *Trichodesmium* spp. is about 34% of *Crocosphaera* spp. Therefore, *Crocosphaera* was the dominant N_2_ fixing cyanobacteria in the study area.

## Additional Information

**How to cite this article**: Kim, D. *et al*. The reduction in the biomass of cyanobacterial N_2_ fixer and the biological pump in the Northwestern Pacific Ocean. *Sci. Rep.* **7**, 41810; doi: 10.1038/srep41810 (2017).

**Publisher's note:** Springer Nature remains neutral with regard to jurisdictional claims in published maps and institutional affiliations.

## Supplementary Material

Supplementary Figure S1 and S2
