# Supplementary material for: The reduction in the biomass of cyanobacterial N2 fixer and the biological pump in the Northwestern Pacific Ocean
Source: Sci Rep. 2017 Feb 3;7:41810. doi: 10.1038/srep41810 (PMC5290482; doi:10.1038/srep41810)
Supplement: Supplementary Figure S1 and S2 [file srep41810-s1.doc]

***Supplementary information***

**The reduction in the biomass of cyanobacterial N_2_ fixer**

**and the biological pump in the Northwestern Pacific Ocean**

Dongseon Kim^1¶^, Jin-Hyun Jeong^1¶^, Tae-Wook Kim^2*^, Jae Hoon Noh^3*^,

Hyung Jeek Kim^4^, Dong Han Choi^3^, Eung Kim^5^, Dongchull Jeon^6^

^1^Marine Chemistry and Geochemistry Research Center, Korea Institute of Ocean Science & Technology, Ansan, South Korea.

^2^Department of Marine Science, Incheon National University, Incheon, South Korea.

^3^Marine Ecosystem and Biological Research Center, Korea Institute of Ocean Science & Technology, Ansan, South Korea.

^4^Deep-sea and Seabed Mineral Resources Research Center, Korea Institute of Ocean Science & Technology, Ansan, South Korea.

^5^Marine Safety Research Center, Korea Institute of Ocean Science & Technology, Ansan, South Korea.

^6^Physical Oceanography Research Center, Korea Institute of Ocean Science & Technology, Ansan, South Korea.

^¶^These authors contributed equally to this work.

*Correspondence and requests for materials should be addressed to T.-W. Kim ([twkim@inu.ac.kr](mailto:twkim@inu.ac.kr), +82-32-835-8878) and J.H. Noh (jhnoh@kiost.ac.kr).

November 8, 2016

To be submitted to SCIENTIFIC REPORTS


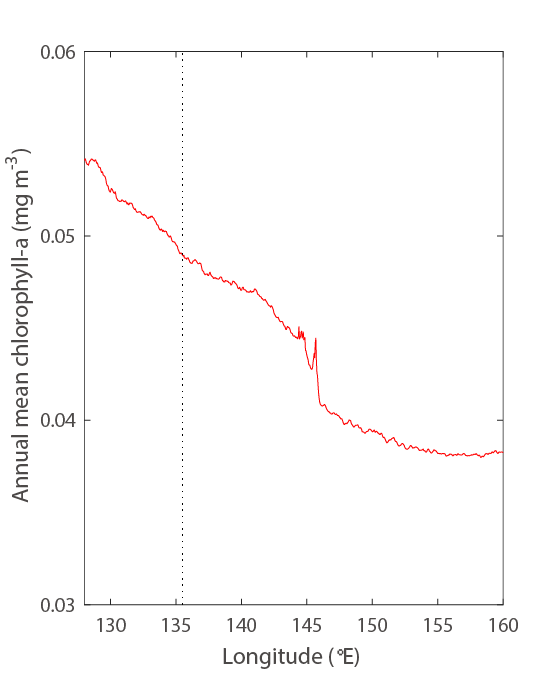


**Figure S1.** Zonal distribution of annual mean chlorophyll-*a* concentrations (mg m^−3^) in the tropical (11.5~15.5°N) Northwestern Pacific Ocean. A dashed line indicates the longitude of the study site. The chlorophyll-*a* concentrations were obtained from the Moderate Resolution Imaging Spectroradiometer (MODIS) aqua dataset.


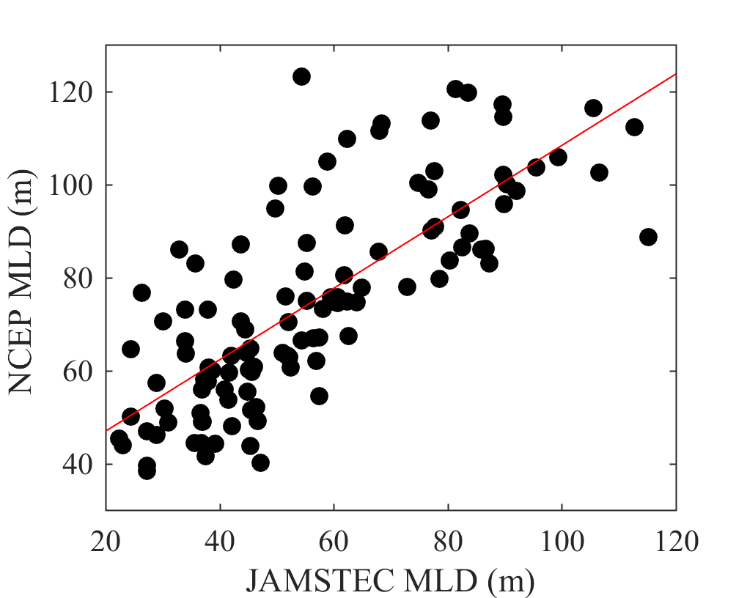


**Figure S2.** Comparison of two independent MLD (mixed layer depth) datasets. Correlation coefficient was 0.76.
